# FLONE: fully Lorentz network embedding for inferring novel drug targets

**DOI:** 10.1093/bioadv/vbad066

**Published:** 2023-05-24

**Authors:** Yang Yue, David McDonald, Luoying Hao, Huangshu Lei, Mark S Butler, Shan He

**Affiliations:** Centre for Computational Biology, School of Computer Science, The University of Birmingham, Edgbaston, Birmingham, B15 2TT, UK; AIA Insights Ltd., 71-75 Shelton Street, London, Greater London, WC2H 9JQ, UK; Centre for Computational Biology, School of Computer Science, The University of Birmingham, Edgbaston, Birmingham, B15 2TT, UK; YaoPharma Co., Ltd., 100 Xingguang Avenue, Renhe Town, Yubei District, Chongqing, 401121, China; AIA Insights Ltd., 71-75 Shelton Street, London, Greater London, WC2H 9JQ, UK; Centre for Computational Biology, School of Computer Science, The University of Birmingham, Edgbaston, Birmingham, B15 2TT, UK

## Abstract

**Motivation:**

To predict drug targets, graph-based machine-learning methods have been widely used to capture the relationships between drug, target and disease entities in drug–disease–target (DDT) networks. However, many methods cannot explicitly consider disease types at inference time and so will predict the same target for a given drug under any disease condition. Meanwhile, DDT networks are usually organized hierarchically carrying interactive relationships between involved entities, but these methods, especially those based on Euclidean embedding cannot fully utilize such topological information, which might lead to sub-optimal results. We hypothesized that, by importing hyperbolic embedding specifically for modeling hierarchical DDT networks, graph-based algorithms could better capture relationships between aforementioned entities, which ultimately improves target prediction performance.

**Results:**

We formulated the target prediction problem as a knowledge graph completion task explicitly considering disease types. We proposed FLONE, a hyperbolic embedding-based method based on capturing hierarchical topological information in DDT networks. The experimental results on two DDT networks showed that by introducing hyperbolic space, FLONE generates more accurate target predictions than its Euclidean counterparts, which supports our hypothesis. We also devised hyperbolic encoders to fuse external domain knowledge, to make FLONE enable handling samples corresponding to previously unseen drugs and targets for more practical scenarios.

**Availability and implementation:**

Source code and dataset information are at: https://github.com/arantir123/DDT_triple_prediction.

**Supplementary information:**

Supplementary data are available at *Bioinformatics Advances* online.

## 1 Introduction

Inferring novel drug targets based on computational methods has attracted more attention recently, because it can effectively reduce the time cost in the early stages of drug development (Oprea *et al.*, 2011). An important systematic strategy for inferring drug targets is based on analyzing known relationships between drug, target and disease entities in biomedical databases, e.g. DrugBank (Wishart *et al.*, 2006) and Pharos (Nguyen *et al.*, 2017). The known relationships in these databases are usually constructed as knowledge graphs (KGs) or heterogeneous biological networks and then are analyzed by graph learning algorithms (Bordes *et al.*, 2013; Grover and Leskovec, 2016). For example, Luo *et al.* compiled their dataset DTINet containing drug–disease–target (DDT) relationships from DrugBank, CTD (Davis *et al.*, 2019), HPRD (Keshava Prasad *et al.*, 2009) and SIDER (Kuhn *et al.*, 2010) databases, and the network diffusion algorithm and inductive matrix completion strategy were further utilized to infer novel drug–target interactions (DTIs) (Luo *et al.*, 2017). Other than the network diffusion-based algorithms, many message passing frameworks based on various information aggregation mechanisms [e.g. attention mechanism (Yu *et al.*, 2021)] have been proposed, to learn the multiple topological characteristics of DDT related networks for interaction predictions between drugs and targets (Chu *et al.*, 2022; Peng *et al.*, 2021; Wan *et al.*, 2019; Wang *et al.*, 2022). In addition, Ye *et al.* generated low-dimensional representations of drugs and targets by integrating their heterogeneous information, extracted from a KG learning method, and their structural information. The final DTIs were predicted by different predictors trained on the produced representations (Ye *et al.*, 2021).

However, many methods usually predict putative associations between drugs and targets (i.e. plain drug–target association predictions), ignoring inferring the relationships between diseases and drugs and between diseases and targets. For example, in the entire DTINet, only 530 out of 5603 diseases have modulation relationships with the target *ADRA1A* (*P35348*) of drug *Clozapine* (*DB00363*). In other words, the target and corresponding given drug could be associated with specific diseases. In this case, explicitly considering disease types when inferring drug targets (i.e. DDT association predictions) could bring finer scale virtual screening compared with plain drug–target or drug–disease association predictions, allowing models to predict drug targets under particular disease types directly. Furthermore, we aimed to examine the model performance in a more realistic application scenario. Specifically, if the users want to discover potential targets for the given drug, they usually need to send every DTI combination corresponding to this drug into the trained model, and rank these DTI combinations according to the scores assigned by the trained model, then the top-ranked DTIs can be selected for further validation experiments (e.g. wet experiments). In this case, the model needs to effectively assign higher ranking scores to the positive target for the given drug and assign relatively lower ranking scores to all other candidate targets for the given drug simultaneously, which is natural to be formulated as a ranking task.

To address these problems, Moon *et al.* (2021) formulated the target prediction as a recommendation system ranking task. The ranking task aims to directly assign each candidate target of interest an interaction score for the given drug and disease. The scores should allow positive targets to rank higher than other negative candidate targets. This ranking task formulation better evaluates the model’s capability to identify positive target samples from all candidate targets, which better reflects true model performance in actual DTI virtual screening. In addition, based on the similar evaluation idea, Chen *et al.* adopted the ranking task to quantify their model capability on predicting drug–target–disease interactions (Chen and Li, 2019; Chen and Li, 2020).

**Fig. 1.**
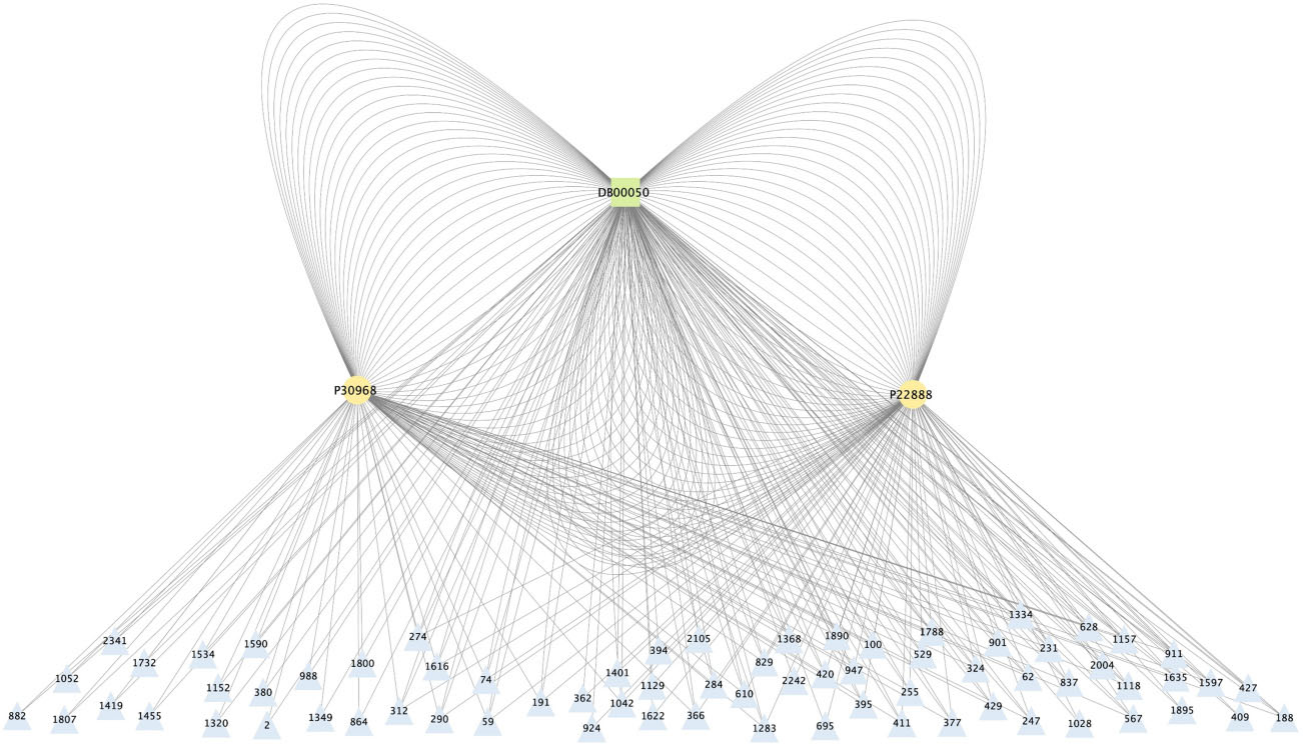
Illustration of the hierarchies in the DDT network. Taking drug node DB00050 in DTINet as an example, it can bind to two target nodes P30968 and P22888 directly, it can also associate with these two targets through 110 different disease nodes based on the drug–disease and disease–target edges (the labels of disease nodes are disease ids used in FLONE). Thus, starting from DB00050, a local hierarchy can be formed (this hierarchy can also be formed starting from the target or disease nodes because these three types of nodes are triangularly interconnected), and such structure can be generalized to other root nodes (in the above example, the root nodes refer to other drugs), forming more complicated implicit hierarchies that reveal the interactive relationships of DDT networks

However, the DDTE method proposed in Moon *et al.* could be further improved by capturing the intrinsic hierarchical structure in DDT networks. As shown in Figure 1, the entity inter-relationships in a DDT network are hierarchically organized. For example, for a drug node *DB00050* in DTINet, which can be seen as a root node, we know it binds to two target nodes *P30968* and *P22888* directly, with which associate 110 different disease nodes based on the drug–disease and disease–target edges. These DDT relationships essentially form a hierarchical structure, which provides extra topological information about the interactive relationships in the DDT network (Corominas-Murtra *et al.*, 2013). Meanwhile, due to the interconnected triangular relationships between drug, target and disease within the network (i.e. the edges can be constructed between these three types of nodes at the same time) (Walsh *et al.*, 2020), such hierarchical structures can also be viewed by treating the target or disease nodes as the root node, while treating the other types of associated nodes as its children nodes. Capturing this hierarchical structural property of the DDT network could be helpful to generate more accurate predictions.

Nevertheless, despite the importance, most graph learning methods including DDTE could not effectively capture this hierarchical information because they work in Euclidean space. It is known that Euclidean space grows polynomially, limiting its capacity to represent hierarchical networks in which the volume of the hierarchies increases exponentially in proportion to its radius (Yang *et al.*, 2022). In contrast, hyperbolic space, which can be seen as a continuous tree space, is a better alternative than Euclidean space since it can fit hierarchies with its exponentially increasing capacity and smaller distortion (Chen *et al.*, 2021). Based on this, we hypothesized that, by properly introducing hyperbolic space, graph machine-learning algorithms could better capture the implicit hierarchies in DDT networks, generating more accurate predictions for each candidate target. To test this hypothesis, we formulated the drug–target prediction as a hyperbolic knowledge graph completion (KGC) task explicitly considering disease types, in which drugs and diseases are subjects and predicates/relations separately, and targets are the objects to be completed (predicted). In this way, KGC can be treated as a (drug, disease, target) triple completion problem, where the drug and disease are given and the target must be inferred.

To solve this problem, we proposed a novel framework, called fully Lorentz network embedding (FLONE), to identify novel targets associated with the given drug and disease, based on utilizing hyperbolic Lorentzian embeddings to learn implicit structural hierarchies of DDT networks. The main component of FLONE is a hyperbolic similarity calculation module based on a fully Lorentz linear transformation (FuLLiT) (Chen *et al.*, 2021). FuLLiT calculates the Lorentzian distance-based similarity probability score between the hyperbolic embeddings of candidate targets and hyperbolic representation of a given drug under a given disease, which is then used to infer a novel target for the drug–disease combination. Another contribution of our work is that, when identifying the DDT triples, the capability to handle previously unseen drug and target entities, which are not included by the seen DDT network, is critical in actual application as not every entity at inference time could be linked with known network structures. To extend FLONE to enable processing such types of entities on the hyperbolic space, we devised our hyperbolic drug and target encoders based on the fully Lorentz linear, linking these unseen entities with the seen entities using drug and target similarity information.

Within the scope of the aforementioned practical application scenario (i.e. testing the model ranking capability based on identifying positive targets from all candidate targets for the given drug and disease), we conducted extensive experiments on FLONE to test our hypothesis. Our study showed that the DDT scoring/ranking benefits from the Lorentz space, which supports our hypothesis. In addition to supporting this hypothesis, our results also showed that by fusing the drug structure and target sequence similarity (as extra domain knowledge), FLONE not only achieved better predictions on DDT triples related to previously seen drugs and targets, but also could provide accurate predictions on the unseen drugs and targets as well.

## 2 Materials and methods

### 2.1 Datasets

To construct the heterogeneous DDT networks based on the DTINet (Luo *et al.*, 2017) and BioKG (Walsh *et al.*, 2020) datasets, we first defined the extraction rule of the DDT triple set. By learning these triples, KGC models can capture the structural property of original DDT networks. We extracted a triple ${(\mathrm{drug}}_{i},{{\mathrm{disease}}_{k},\mathrm{target}}_{j})$ [abbreviated as ${(D}_{i},{D_{k}^{\prime },T}_{j}$)] if all three of the following edges exist in the original dataset: ${(D}_{i},D_{k}^{\prime }$), ${(D}_{i},T_{j}$) and ${{(D}_{k}^{\prime },T}_{j}$).

However, in KGC tasks, there could be implicit data leakage caused by very similar predicates, e.g. for two (subject, predicate, object) triples, (‘Birmingham’, ‘is_in’, ‘UK’) and (‘Birmingham’, ‘is_located_in’, ‘UK’), because the predicates 'is_in' and 'is_located_in' have very similar semantic meaning, thus they correspond to very similar subject–object pairs, which causes over-idealistic results when predicting triples related to 'is_in' or 'is_located_in'. To avoid this problem, we removed diseases that had >60% drug–target pair similarity with other diseases based on the Jaccard similarity coefficient that can measure the similarity of different (drug–target pair) sets (detailed in Supplementary Section S1). After the screening, 171 597 positive triples consisting of 535 drugs, 417 targets and 1160 diseases from DTINet as well as 9699 positive triples consisting of 1128 drugs, 723 targets and 529 diseases from BioKG remained. However, inappropriate data splitting for model evaluation will lead to another type of data leakage, which is detailed in Section 3.2. In addition, the detailed description of used drug and target similarity information for handling network learning unseen drugs and targets are provided in Section 2.5.

### 2.2 The basic definition of the Lorentz model

The hyperbolic space is defined as a smooth Riemannian manifold equipped with the constant negative curvature and positive-definite inner product on the tangent space at every point (Yang *et al.*, 2022). There are several isomorphic geometric models of the hyperbolic space: Lorentz model, Poincaré disk model, Poincaré half-plane model and Klein model. In this article, we used the Lorentz model, which is one of the widely used models because of its numerical stability and closed-form computation of geodesics (Chen *et al.*, 2021; McDonald and He, 2022; Sun *et al.*, 2021; Wu *et al.*, 2021; Yu *et al.*, 2020).

The n-dimensional Lorentz model with curvature c (-1 in our study) is defined as $L_{c}^{n}=(L^{n},g_{x}^{c})$, $g_{x}^{c}=\eta$ is the Riemannian metric tensor satisfying η=I [I is the (n+1)-dimension diagonal matrix] except $\eta_{0,0}=-1$, and $L^{n}$ represents the manifold defined by the point set in $L_{c}^{n}$:


(1) $$L^{n}≔\left\{x\in R^{n+1}:{〈x,\;x〉}_{L}=\frac{1}{c},x_{t}>0\right\},$$

(2) $${〈x,\;y〉}_{L}≔x^{T}g_{x}^{c}y=-x_{t}y_{t}+x_{s}^{T}y_{s},$$

where ${〈\ldots ,\;\ldots 〉}_{L}$ is the Lorentzian inner product. Each point x in $L_{c}^{n}$ is expressed as a concatenation $\left[x_{t},\;x_{s}\right]$, where $x_{t}\in R$ (referred as the time dimension), $x_{s}\in R^{n}$ (referred as the spatial dimension) and the origin O of the Lorentz model is defined as $\left(\sqrt{-1/c},0,\ldots ,0\right)$. In other words, x represents the space coordinate point in the defined n-dimensional Lorentz space, all drugs and targets will be embedded into this Lorentz space, and the allocated coordinate points/positions of drugs and targets can be seen as their feature embeddings. The n-dimensional Lorentz space consists one temporal dimension and n spatial dimensions, which are used to depict the coordinate position/embedding feature on each dimension here. For further understanding, we suggested referring (Yang *et al.*, 2022) for a more detailed description.

Besides, for every point $x\in L_{c}^{n}$, it is equipped with an orthogonal space (i.e. tangent space) of $L_{c}^{n}$ (at x), which is the first-order approximation of $L^{n}$ around x (Yang *et al.*, 2022), and is formally defined as $T_{x}L_{c}^{n}≔\{z\in R^{n+1}:{〈z,\;x〉}_{L}=0\}$, where z is the point set of this tangent space.

### 2.3 Description of the FLONE method

Based on the defined Lorentz model, we proposed FLONE to solve the DDT triple target entity completion problem. Specifically, for each DDT triple in the extracted triple set, FLONE treats the drug, disease and target as the subject, predicate/relation and object, respectively. The task is, given a drug–disease combination, FLONE will assign a similarity score to every candidate target (i.e. object entity) in the DDT network, which indicates the distance-based similarity between the target and given drug and disease. FLONE then uses the similarity scores to rank these targets, for identifying high-confidence targets for the drug–disease combination.

A high-quality model would assign high similarity scores to all of the defined DDT triples that have been extracted from the DDT network, and low similarities for all other DDT combinations. A researcher investigating drug repurposing would provide the drug and disease of interest and will have a ranked list of targets returned for further investigation.

**Fig. 2.**
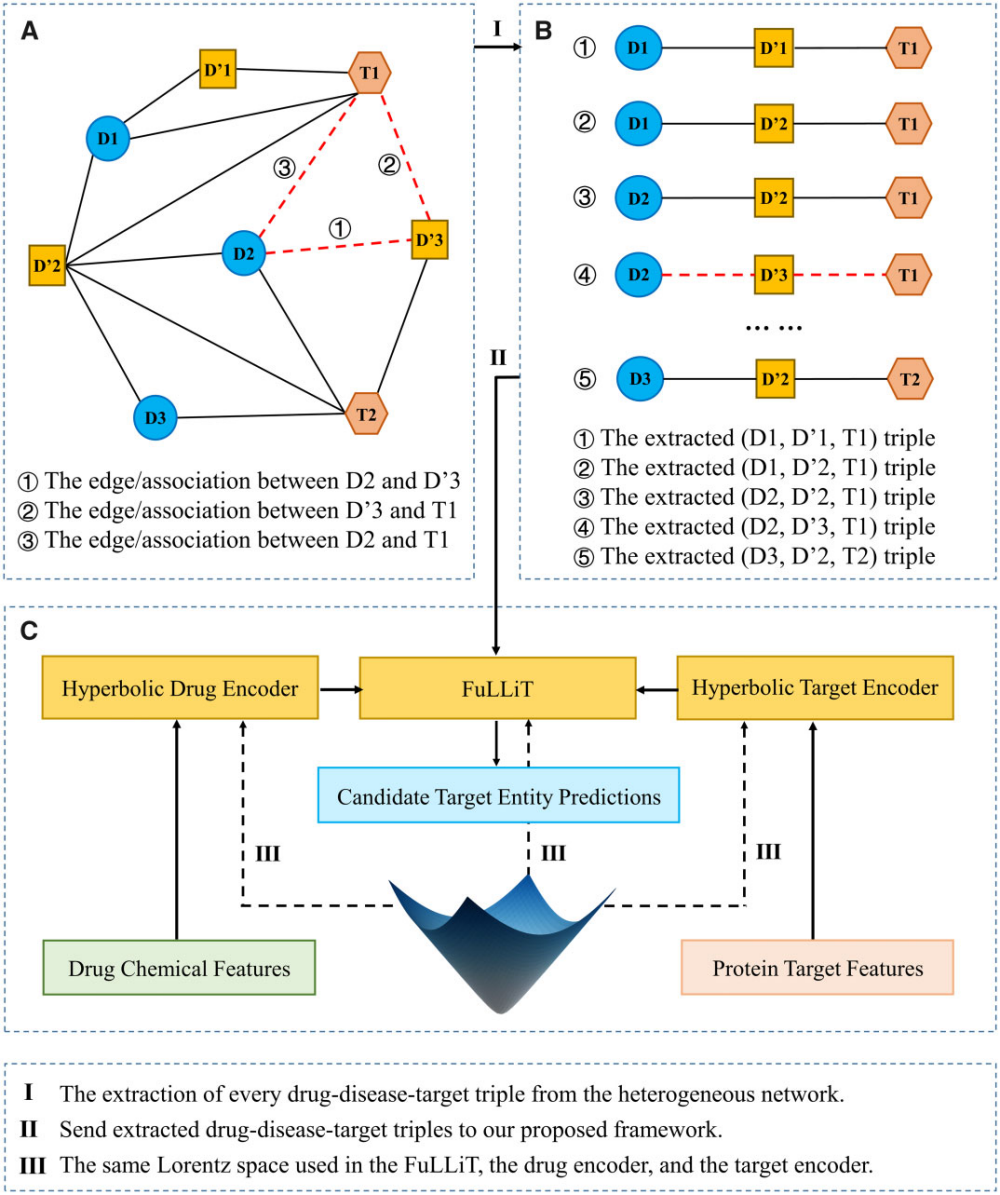
Illustration of FLONE. (**A**) Construct a heterogeneous DDT network. (**B**) Decompose this network to extract the DDT triple set. (**C**) Input the Euclidean drug and target (similarity) domain knowledge into the hyperbolic drug and target encoders, respectively, to generate hyperbolic (Lorentzian) drug and target embeddings (or using self-contained drug and target embeddings in FuLLiT directly). Next, the hyperbolic drug and target embeddings are used by FuLLiT, in which hyperbolic drug embeddings are transformed by the disease translation representations, and then the Lorentzian distance-based similarity between the transformed drug embeddings and hyperbolic target embeddings are calculated. After training, FLONE is able to rank every candidate target entity for the given drug and disease

The illustration of FLONE is shown in Figure 2. After the extraction of triples from the heterogeneous DDT network, we presented FLONE as an end-to-end framework consisting of three major components:

1. FuLLiT, a feed-forward neural network operating within Lorentzian space, which was proposed to increase the performance of the Euclidean translation-based KGC method in our task. FuLLiT treats a (disease) predicate/relation as a trainable translation offset from the (drug) subject to the (target) object entity embeddings. It can take two n-dimensional Lorentzian vectors, representing a drug embedding and a target embedding, along with a disease index as inputs, and outputs a similarity score for the DDT. FuLLiT is detailed in Section 2.4.
2. Hyperbolic drug encoder, which is responsible for taking drug chemical features to produce a hyperbolic Lorentzian embedding for a drug. It can inject drug domain knowledge into FuLLiT for handling previously unseen drugs at inference time (explained in more detail in Section 2.5).
3. Hyperbolic target encoder, which, likewise, produces hyperbolic Lorentzian embeddings for all candidate targets, to fuse target domain knowledge into FuLLiT for handling unseen targets (detailed in Section 2.5).

In addition, all important hyperbolic computation operations of FLONE are fully Lorentzian instead of a hybrid mode (Chen *et al.*, 2021). This has the advantage that there is no need to expensively map between the Lorentz space and its tangent space (McDonald and He, 2022).

### 2.4 Description of FuLLiT

Based on the fully Lorentz linear layer, FuLLiT was developed, which includes three components: (i) a hyperbolic triple decoder, (ii) a self-contained drug embedding look-up table and (iii) a self-contained target embedding look-up table. The first component is to calculate the similarity score between the target and given drug and disease for each DDT triple, and the latter two are for providing corresponding hyperbolic embeddings without drug/target similarity information (i.e. only utilizing structural information of DDT networks). These three components form the backbone of FLONE that can deal with cases where external domain knowledge is unavailable.

The general form of the fully Lorentz linear layer ${\mathrm{FLLinear}}_{n,m}(x)$ (contained in FuLLiT) is defined as (3), which could ensure a linear transformation to map $x\in L_{c}^{n}$ ($x\in R^{n+1}$) to $y\in L_{c}^{m}$ ($y\in R^{m+1}$) (i.e. the coordinates of input x and output y of the transformation are guaranteed to stay in the respective Lorentz space): where λ is a fixed hyper-parameter to control the numerical scale of the time dimension, σ is the sigmoid function, $v\in R^{n+1}$ and $W\in R^{m\times (n+1)}$ are trainable weights of the overall linear transformation matrix $M=[v^{T}/W]\;$([…/…] represents the tensor concatenation) in this linear layer. Besides, b is the trainable bias, ϵ is a fixed value larger than $\sqrt{-1/c}$ to ensure $\sqrt{{\left(\lambda \sigma \left(v^{T}x+b\right)+\epsilon \right)}^{2}+1/c}$ larger than 0. In FLONE, we adopted the same dimension for all embeddings, in this case, n is equal to m in every intermediate ${\mathrm{FLLinear}}_{n,m}(x)$ layer.


$$y={\mathrm{FLLinear}}_{n,m}(x)$$

(3) $$=\frac{\sqrt{{\left(\lambda \sigma \left(v^{T}x+b\right)+\epsilon \right)}^{2}+\frac{1}{c}}}{∥W\left(\mathrm{dropout}\left(x\right)\right)∥}W\left(\mathrm{dropout}\left(x\right)\right),$$

For the hyperbolic triple decoder of FuLLiT, assuming embedding all entities into a n-dimensional Lorentz space, the following steps are included to calculate the similarity scores (from 0 to 1). Take an example of predicting the score for $T_{j}$ under $\left(D_{i},D_{k}^{\prime }\right)$, five sets of parameters are needed, including corresponding drug and target embeddings, disease type-specific translation offset (for $D_{k}^{\prime }$) and real-value drug and target biases. Specifically, we first obtained the hyperbolic embedding $x_{D_{i}}\in L_{c}^{n}$ and $x_{T_{j}}\in L_{c}^{n}$ of $D_{i}$ and $T_{j}$ from the self-contained embedding look-up tables (i.e. the tables that store the corresponding type of embeddings through entity indices). Then, $x_{D_{i}}\in L_{c}^{n}$ was transformed by the disease type-specific translation offset ${\mathrm{FLLinear}}_{n,n}\left(x\right)$ (for $D_{k}^{\prime }$), to obtain the translation $x_{D_{i}-D_{k}^{\prime }}\in L_{c}^{n}$. Then the similarity score p can be calculated as (Balazevic *et al.*, 2019; Chen *et al.*, 2021): where $d_{L}^{2}\left(x_{D_{i}-D_{k}^{'}},x_{T_{j}}\right)$ represents the squared Lorentzian distance measuring the similarity between the given drug–disease combination and corresponding target (Law *et al.*, 2019; Ratcliffe, 2006), $b_{D_{i}}$ and $b_{T_{j}}$ are the drug and target type-specific biases, respectively. φ is the margin hyper-parameter, and σ is the sigmoid function.


(4) $$p\left(D_{i},D_{k}^{\prime },T_{j}\right)=\sigma \left(-d_{L}^{2}\left(x_{D_{i}-D_{k}^{\prime }},x_{T_{j}}\right)+b_{D_{i}}+b_{T_{j}}+\varphi \right),$$

(5) $$d_{L}^{2}\left(x_{D_{i}-D_{k}^{\prime }},x_{T_{j}}\right)=\frac{2}{c}-2{〈x_{D_{i}-D_{k}^{\prime }},\;x_{T_{j}}〉}_{L},$$

As for the above self-contained drug and target embedding look-up tables, they are essentially trainable matrices formally defined as drug_lookup and target_lookup with the shapes of $\left(\mathrm{drug}\;\mathrm{number},\;n\right)$ and $\left(\mathrm{target}\;\mathrm{number},\;n\right)$, in which n is the overall feature dimension of the Lorentz model, drug number and target number are the number of all drugs of interest and all candidate targets, respectively:


(6) $$\begin{array}{c} x_{D_{i}}\in L_{c}^{n}\leftarrow \text{drug}\_\text{lookup}\left(D_{i}\right)\\ \;x_{T_{j}}\in L_{c}^{n}\leftarrow \text{target}\_\text{lookup}\left(T_{j}\right). \end{array}$$

Specifically, the shape of the look-up tables is determined based on the number of drugs and targets under which we would like to explore the potential drug targets. They can be trained together with other sets of parameters, to produce the required hyperbolic embeddings based on learning topological structural information of DDT networks. Besides, the look-up tables can also be created by hyperbolic drug and target encoders (detailed in the next section) in an end-to-end way (all sets of parameters are learnt simultaneously based on known triples extracted from DDT networks), for injecting external domain knowledge to handle the unseen drugs and targets.

As a comparison, the Euclidean translation-based method DDTE (Moon *et al.*, 2021) mentioned in Introduction, uses vectors as the disease translation offsets. Specifically, it used vector-based disease offset $V_{D_{k}^{\prime }}$ to calculate the translated drug embedding under the given disease (i.e. $e_{D_{i}}+V_{D_{k}^{\prime }}$, $e_{D_{i}}$ is the Euclidean drug embedding). While for FuLLiT, it was calculated based on the matrix-based offset ${\mathrm{FLLinear}}_{n,n}\left(x_{D_{i}}\right)$. Except for specialized hyperbolic operations, another two main differences between DDTE and FuLLiT/FLONE are that compared with FLONE, when calculating the similarity score, there are no drug and target-specific biases in Equation (4) for DDTE, and DDTE cannot consider external domain knowledge (i.e. only providing Euclidean self-contained drug and target look-up tables).

### 2.5 The hyperbolic encoders for fusing drug and target similarity information

To investigate whether the triple target entity completion benefits from the fusion of domain knowledge, we extended the FuLLiT, by replacing the self-contained drug and target embedding look-up tables with the tables generated by corresponding hyperbolic encoders, for encoding and injecting external domain knowledge into embeddings, and the size of these embeddings is same as that in the self-contained look-up tables.

Specifically, we tried to fuse different domain knowledge of drug–drug and target–target similarities by utilizing hyperbolic drug and target encoders. Intuitively, this could bring similar hyperbolic embeddings for involved similar drugs/targets. Based on the assumption ‘similar drugs may share similar targets and vice versa’ (Luo *et al.*, 2017), this enables the further predictive performance improvement of FLONE.

For the drugs of interest, we computed Extended Connectivity Fingerprints of diameter 6 (ECFP6), a circular topological fingerprint commonly used in drug discovery (Rogers and Hahn, 2010), as the input of the hyperbolic drug encoder. For the involved targets, we provided target sequence similarity (i.e. protein similarity scores based on primary sequences of target proteins after 0–1 normalization) (Luo *et al.*, 2017), for the hyperbolic target encoder.

Since all numerical operations in FLONE are defined on Lorentz space, these (Euclidean) drug and target features must first be mapped to vectors on the Lorentzian manifold $L^{n}≔\{x\in R^{n+1}:{〈x,\;x〉}_{L}=1/c,x_{t}>0\}$. To this end, the tangent space of Lorentz space $T_{x}L_{c}^{n}≔\{z\in R^{n+1}:{〈z,\;x〉}_{L}=0\}$ is needed for the mapping. Specifically, for the hyperbolic drug encoder, it takes the (Euclidean) ECFP6 as the input. Take encoding ECFP6 of $D_{i}$ (termed as ${\mathrm{ECFP}}_{D_{i}}$) as an example, first, ${\mathrm{ECFP}}_{D_{i}}$ is concatenated with 0 to create (0, ${\mathrm{ECFP}}_{D_{i}}$), to map ${\mathrm{ECFP}}_{D_{i}}$ into the tangent space of origin of Lorentz space $O=(\sqrt{-1/c},0,\ldots ,0)$. Because according to $T_{x}L_{c}^{n}≔\{z\in R^{n+1}:{〈z,\;x〉}_{L}=0\}$ and ${〈(0,\;{\mathrm{ECFP}}_{D_{i}}),\;O〉}_{L}=0$, (0, ${\mathrm{ECFP}}_{D_{i}}$) is in the tangent space of O. Then, (0, ${\mathrm{ECFP}}_{D_{i}}$) is further mapped into the Lorentz space through the exponential map function defined as follows, where $z\in T_{x}L_{c}^{n}$:


(7) $$\exp_{x}^{c}\left(z\right)=\cosh\left(\sqrt{\left|c\right|}{\left\|z\right\|}_{L}\right)x+z\frac{\sinh(\sqrt{\left|c\right|}{\left\|z\right\|}_{L})}{\sqrt{\left|c\right|}{\left\|z\right\|}_{L}},$$

(8) $${\left\|z\right\|}_{L}=\sqrt{{〈z,\;z〉}_{L}}.$$

In this way, the Lorentzian ECFP6, i.e. $\exp_{O}^{c}\left(\left(0,\;{\mathrm{ECFP}}_{D_{i}}\right)\right)$ can be generated, and then $\exp_{O}^{c}\left(\left(0,\;{\mathrm{ECFP}}_{D_{i}}\right)\right)$ is sent to ${\mathrm{FLLinea}r}_{m,n}\left(x\right)$ specifically for encoding Lorentzian ECFP6, to reduce its dimension to the unified hidden dimension mentioned in Section 2.4. Furthermore, all Lorentzian ECFP6 (after dimension reduction) of involved drugs constitutes the hyperbolic drug embedding look-up table (through drug entity indices). As an analogy (above procedure can be used to map any arbitrary Euclidean vector to a Lorentzian vector), based on the similar procedure with a ${\mathrm{FLLinear}}_{m,n}\left(x\right)$ specifically for target sequence similarity, the hyperbolic target encoder can be generated.

After combining the hyperbolic encoders with FuLLiT, the parameters to be end-to-end optimized are not the weight of self-contained trainable matrices anymore but different weights in fully Lorentz layers of the encoders. The advantage of adding domain knowledge-based encoders is that, previously unseen drugs and targets can be handled by linking them with seen entities with the help of the injected prior similarity.

### 2.6 Model training and optimization

Similar to the Euclidean translation-based KGC models, negative sampling (Bordes *et al.*, 2013) was used to train the FLONE. Specifically, during the training phase, for each positive/known triple $\left(D_{i},{D_{k}^{\prime },T}_{j}\right)$, the negative triple was sampled by randomly replacing $T_{j}$ with other $T_{j^{\prime }}$ in the target entity set and ensuring that the generated negative triples were not in the extracted known DDT triple set.

The loss function used for optimizing all sets of parameters in FLONE was the binary cross entropy loss defined as follows: where N is the positive/known triple number in the training set and $N^{\prime }$ is the number of negative triples generated for each known triple. Besides, we adopted the Riemannian Adam as the optimizer of FLONE, which is the counterpart of Adam defined in Euclidean space (Bécigneul and Ganea, 2018; Kochurov *et al.*, 2020).


(9) $$L=-\frac{1}{N}\sum^{N}\left(\log p\left(D_{i},D_{k}^{\prime },T_{j}\right)+\sum^{N^{\prime }}\log\left(1-p\left(D_{i},D_{k}^{\prime },T_{j^{\prime }}\right)\right)\right),$$

## 3 Results and discussion

### 3.1 Hyperbolicity of the DDT networks and scalability analysis

To test whether the resulting heterogeneous DDT networks (equivalent to the extracted triple sets) exhibit a hierarchical structure for demonstrating our hypothesis, we calculated their Gromovs hyperbolicity δ (Chami *et al.*, 2019; Gromov, 1987), which measures how hierarchical the network is. The lower δ, the more implicit hierarchies the network has, and δ of completely tree-like structures is 0. Moreover, for common hierarchical benchmark datasets, e.g. Human PPI and Airport, the δ is about 1, and for the standard (non-hierarchical) benchmark, e.g. Cora, the δ is 11 (Chami *et al.*, 2019). While the calculated δ values of the extracted DTINet and BioKG DDT networks were both 1.5, which indicated that these networks do possess the implicit hierarchies, making them theoretically feasible for hyperbolic space embedding.

The model complexity of FLONE is $O(dn_{e}+n_{e}+n_{r}d^{2})$, in which d, $n_{e}$ and $n_{r}$ are the embedding dimension, (drug and target) entity number and (disease) relation number, respectively. Specifically, the complexity of either self-contained look-up tables or hyperbolic encoders based on ${\mathrm{FLLinear}}_{m,n}\left(x\right)$ ($O(dn_{e})$) is proportional to d and $n_{e}$ and irrelevant to $n_{r}$, as the matrix contained in them is independent of disease types. Besides, as demonstrated by Chen *et al.* (2021), the fully Lorentzian-based KGC algorithm can be effectively extended to KG triple datasets with over 40 000 entity number and hundreds of relation types (Dettmers *et al.*, 2018; Toutanova and Chen, 2015). To the best of our knowledge, this scale can satisfy handling most of the precisely curated DDT triple sets.

### 3.2 Model evaluation settings

Except for the data leakage mentioned in Section 2.1, the common model evaluation setting, i.e. randomly splitting the DDT triple set into training, validation, and test sets could also cause over-idealistic results in our task. This is because each defined known $\left(D_{i},{D_{k}^{\prime },T}_{j}\right)$ triple not only contains the associations $\left(D_{i},D_{k}^{\prime }\right)$ and ${{(D}_{k}^{\prime },T}_{j})$, but also includes $\left(D_{i},T_{j}\right)$ (i.e. constituting a triangular inter-node sub-structure), and ignoring ${(D}_{i},T_{j})$ in data splitting will lead to extra data leakage. For example, if $\left(D_{i},{D_{k1}^{\prime },T}_{j}\right)$ and ${(D}_{i},{D_{k2}^{\prime },T}_{j})$ are allocated into training and test sets separately, after training, when inferring the target for $\left(D_{i},D_{k2}^{\prime }\right)$, $T_{j}$ tends to be chosen more easily, as the model ‘has already seen’ the unnecessary implicit association information between $D_{i}$ and $T_{j}$ [i.e. ${(D}_{i},T_{j})$] during the training phase.

To avoid this pitfall, we split these triples into training, validation and test sets based on drug–target pairs. In other words, the known triples with the same drug–target pair were put into the same set, as such all test targets for a given drug were not seen by the model during training. In this case, no consideration was given to ensure a complete coverage of drugs and targets in the training set, and so it was possible for drugs and targets to exist in either the validation or test set, but not in the training set (the related statistics are in Supplementary Section S4), which also increased the difficulty of target identifications.

Based on this setting, the DDT triples corresponding to 60%: 20%: 20% of all drug–target pair varieties were divided into training, validation and test sets, respectively. This procedure was repeated five times independently, for each time, before splitting data, the whole DDT triple set was randomly shuffled to make different drug–target pair varieties enter each set. We computed and reported the average evaluation metrics over the five independent repeats.

To evaluate the model’s predictive performance in the scenario of explicitly considering disease types and directly ranking all candidate targets, we adopted the standard evaluation metrics used in recommendation system ranking tasks, including Mean Reciprocal Rank (MRR), Hits@1, Hits@3 and Hits@10 (Moon *et al.*, 2021). Among these, MRR was chosen as the main metric, because it can better evaluate the model’s ability to assign the positive target a ranking score that is distinguishable from other candidate targets (under the given drug and disease). MRR is calculated as follows: where $N_{\mathrm{test}}$ is the test sample set, and ${\mathrm{rank}}_{i}$ is the score ranking of the true target entity among all targets of interest for the $i_{\mathrm{th}}$ test sample. Hits@K represents the percentage of the true target entities that appear within the top K positions of overall candidate ranking during the test phase.


(10) $$\mathrm{MRR}=\frac{1}{\left|N_{\mathrm{test}}\right|}\sum_{i=1}^{\left|N_{\mathrm{test}}\right|}\frac{1}{{\mathrm{rank}}_{i}},$$

### 3.3 Experiments without external domain knowledge injection

The main objective of our experiments is to test whether properly introducing hyperbolic space can improve the performance of Euclidean translation-based KGC methods in our task. To this end, we compared our method with the representative Euclidean translation-based method DDTE detailed in Section 2.4 based on the aforementioned data splitting. To eliminate the uncontrolled influence brought by different external domain knowledge for fair comparison, we first chose FuLLiT (termed as ${\mathrm{FLONE}}_{\mathrm{base}}$, later these two names will be used interchangeably), which only uses self-contained drug and target embedding look-up tables (the performance of FLONE with the hyperbolic encoder under the same experimental settings/data splitting was discussed in the next section). The two additional algorithm variants were also considered. The first variant was named as ${\mathrm{DDTE}}_{\mathrm{bias}}$, in which the drug and target type-specific biases mentioned in Equation (4) were added into DDTE. The second variant was the fully Euclidean counterpart of ${\mathrm{FLONE}}_{\mathrm{base}}$, termed as ${\mathrm{FEC}-\mathrm{FLONE}}_{\mathrm{base}}$: on the top of ${\mathrm{DDTE}}_{\mathrm{bias}}$, the vector-based disease offset was replaced by the matrix-based offset [i.e. using ${\mathrm{ELinear}}_{n,n}\left(e_{D_{i}}\right)$ as the offset, where ${\mathrm{ELinear}}_{n,n}$ and $e_{D_{i}}$ represent the Euclidean linear layer and Euclidean drug embedding separately]. This controls for the internal representation of diseases by representing diseases as translation matrices in both algorithms.

To conduct more comprehensive comparison, we also considered two other Euclidean KGC methods ConvE (Dettmers *et al.*, 2018) (Convolution-based) and DistMult (bilinear product-based) (Yang *et al.*, 2014) as well as another hyperbolic method MuRP (Poincaré space-based) (Balazevic *et al.*, 2019). Further, we did experiments based on two embedding dimensions: 16 and 128. Sixteen and one hundred twenty-eight are commonly selected, representative dimensions for hyperbolic and Euclidean embedding separately (Chen *et al.*, 2021; Moon *et al.*, 2021). Theoretically, hyperbolic embedding loses less information than Euclidean embedding when the embedding dimension is small. Besides, the Adam optimizer (Kingma and Ba, 2014) was adopted for all Euclidean-based models.

The experimental results are in Tables 1 and 2. Both dimensions suggest that ${\mathrm{FLONE}}_{\mathrm{base}}$ achieved overall better performance compared with all involved Euclidean-based models across both embedding dimensions and on both the DTINet and BioKG datasets. Only under Hits@10, the sub-optimal performance was obtained on DTINet. Compared with the second-best model (based on the main metric MRR), ${\mathrm{FLONE}}_{\mathrm{base}}$ obtained 5.5% and 5.8% (on DTINet) and 8.0% and 5.7% (on BioKG) performance margins on the 16-dimension and 128-dimension, respectively. The above clearly verified the effectiveness of FLONE and our hypothesis. Interestingly, MuRP did not produce very close results with our Lorentz space-based method due to numerical instability of the Poincaré model, suggesting our decision to use the Lorentz model.

**Table 1.** Comparison results of involved methods under the 16 embedding dimension

DTINet	MRR	Hits@1	Hits@3	Hits@10
${\mathrm{FLONE}}_{\mathrm{base}}$	**0.3806**	**0.3301**	**0.3994**	0.4685
${\mathrm{FEC}-\mathrm{FLONE}}_{\mathrm{base}}$	0.3608	0.3174	0.3680	0.4484
${\mathrm{DDTE}}_{\mathrm{bias}}$	0.3134	0.2315	0.3520	0.4651
DDTE	0.3049	0.2123	0.3518	**0.4790**
MuRP	0.3234	0.2463	0.3605	0.4636
ConvE	0.3160	0.2362	0.3583	0.4648
DistMult	0.3014	0.2148	0.3387	0.4739

BioKG	MRR	Hits@1	Hits@3	Hits@10

${\mathrm{FLONE}}_{\mathrm{base}}$	**0.4706**	**0.3786**	**0.5224**	**0.6452**
${\mathrm{FEC}-\mathrm{FLONE}}_{\mathrm{base}}$	0.3594	0.3049	0.3797	0.4600
${\mathrm{DDTE}}_{\mathrm{bias}}$	0.4222	0.3302	0.4653	0.5992
DDTE	0.3545	0.2417	0.4162	0.5586
MuRP	0.4358	0.3373	0.4911	0.6146
ConvE	0.3737	0.2815	0.4182	0.5507
DistMult	0.2877	0.1986	0.3310	0.4518

*Note*: The bold data indicate the best result under current evaluation metric and dataset.

**Table 2.** Comparison results of involved methods under the 128 embedding dimension

DTINet	MRR	Hits@1	Hits@3	Hits@10
${\mathrm{FLONE}}_{\mathrm{base}}$	**0.4335**	**0.3936**	**0.4428**	0.5054
${\mathrm{FEC}-\mathrm{FLONE}}_{\mathrm{base}}$	0.4098	0.3705	0.4139	0.4907
${\mathrm{DDTE}}_{\mathrm{bias}}$	0.3893	0.3232	0.4165	**0.5125**
DDTE	0.3139	0.2186	0.3635	0.4997
MuRP	0.3310	0.2502	0.3699	0.4823
ConvE	0.3885	0.3389	0.4090	0.4702
DistMult	0.3555	0.3000	0.3780	0.4536

BioKG	MRR	Hits@1	Hits@3	Hits@10

${\mathrm{FLONE}}_{\mathrm{base}}$	**0.5027**	**0.4116**	**0.5495**	**0.6758**
${\mathrm{FEC}-\mathrm{FLONE}}_{\mathrm{base}}$	0.4549	0.3790	0.4914	0.5960
${\mathrm{DDTE}}_{\mathrm{bias}}$	0.4754	0.3780	0.5215	0.6671
DDTE	0.3806	0.2549	0.4576	0.6071
MuRP	0.4750	0.3749	0.5323	0.6586
ConvE	0.4641	0.3781	0.5090	0.6296
DistMult	0.3863	0.3176	0.4198	0.5108

*Note*: The bold data indicate the best result under current evaluation metric and dataset.

### 3.4 Fusing domain knowledge for FLONE

After demonstrating our basic hypothesis, following the same experimental settings/data splitting as the last section, we investigated the effectiveness of fusing domain knowledge for FLONE. As described in Section 3.2, current data splitting is based on a challenging scenario where previously unseen drugs and targets exist. It is difficult for FuLLiT and DDTE to handle the included unseen drugs and targets because what they leverage is only known structural information of the DDT network. Intuitively, the fusion of similarity domain knowledge of involved drugs and targets could be helpful to improve the prediction accuracy of FuLLiT. To demonstrate this, based on the larger and more complex DTINet DDT network with complete similarity data, the FLONE variants with different hyperbolic drug and target encoder combinations for FuLLiT (i.e. ${\mathrm{FLONE}}_{\mathrm{ECFP}-\mathrm{SEQ}}$, ${\mathrm{FLONE}}_{\mathrm{ECFP}-\mathrm{None}}$ and ${\mathrm{FLONE}}_{\mathrm{None}-\mathrm{SEQ}}$), were added into performance comparison. The two suffixes (first: drugs, second: targets) of the variant name represent the use of the corresponding encoders (ECFP: hyperbolic ECFP6-based drug encoder, SEQ: hyperbolic target sequence similarity-based target encoder and None: using the original self-contained embedding look-up table without domain knowledge injection). Additionally, we compared two representative network-based target prediction methods NeoDTI (Wan *et al.*, 2019) and KGE_RF (Ye *et al.*, 2021), which were trained based on a standard 1:1 sampling of positive and negative samples (the detailed experimental setup of these two methods are in Supplementary Section S2). Since the final embedding dimension of KGE_RF depends on the selected drug and target structural information dimension, we adopted their default basic hyper-parameters.

Table 3 gives the predictive performance of all of the aforementioned algorithms under the experimental setting described in Section 3.2. From the results, we first found that, compared with the other FLONE variants, ${\mathrm{FLONE}}_{\mathrm{ECFP}-\mathrm{SEQ}}$ had the best performance. Under both 16 and 128 dimensions, it obtained 24.5% and 30.2% performance improvements on MRR compared with ${\mathrm{FLONE}}_{\mathrm{base}}$/FuLLiT. This can demonstrate the importance of similarity domain knowledge in current scenario where unseen drugs and targets occur. ${\mathrm{FLONE}}_{\mathrm{ECFP}-\mathrm{None}}$ was better than ${\mathrm{FLONE}}_{\mathrm{None}-\mathrm{SEQ}}$, indicating that ECFP6 could be more effective compared with the target sequence similarity in our task. In addition, ${\mathrm{FLONE}}_{\mathrm{ECFP}-\mathrm{SEQ}}$ also outperformed the involved NeoDTI and KGE_RF algorithms, which further demonstrated the effectiveness of our framework. Meanwhile, we provided the experiments about fusing similarity domain knowledge into the Euclidean-based models in Supplementary Section S3.

**Table 3.** Comparison results of involved methods under the 16/128 embedding dimensions based on the DTINet dataset

Methods	MRR	Hits@1	Hits@3	Hits@10
${\mathrm{FLONE}}_{\mathrm{ECFP}-\mathrm{SEQ}}\;$ dim: 16	**0.4740**	**0.4121**	**0.4945**	**0.5946**
${\mathrm{FLONE}}_{\mathrm{ECFP}-\mathrm{None}}$ dim: 16	0.4322	0.3750	0.4568	0.5347
${\mathrm{FLONE}}_{\mathrm{None}-\mathrm{SEQ}}$ dim: 16	0.4068	0.3461	0.4233	0.5260
${\mathrm{FLONE}}_{\mathrm{base}}$ dim: 16	0.3806	0.3301	0.3994	0.4685
NeoDTI dim: 16	0.1464	0.0539	0.1563	0.3245

${\mathrm{FLONE}}_{\mathrm{ECFP}-\mathrm{SEQ}}$ dim: 128	**0.5644**	**0.5139**	**0.5793**	**0.6564**
${\mathrm{FLONE}}_{\mathrm{ECFP}-\mathrm{None}}\;$ dim: 128	0.5033	0.4571	0.5216	0.5912
${\mathrm{FLONE}}_{\mathrm{None}-\mathrm{SEQ}}$ dim: 128	0.4820	0.4410	0.4892	0.5614
${\mathrm{FLONE}}_{\mathrm{base}}$ dim: 128	0.4335	0.3936	0.4428	0.5054
NeoDTI dim: 128	0.1981	0.1051	0.1961	0.4054

KGE_RF	0.2761	0.1859	0.3081	0.4679

*Note*: The bold data indicate the best result under current evaluation metric.

### 3.5 Extra ablation study

Based on the conclusion from the last section, to confirm that the performance gain seen in FLONE having drug and target encoders compared with the one without the encoders, is not solely due to the later one’s inability to handle unseen drugs and targets, we devised an extra ablation study: we still kept the same experimental settings, to run FuLLiT/${\mathrm{FLONE}}_{\mathrm{base}}$ and ${\mathrm{FLONE}}_{\mathrm{ECFP}-\mathrm{SEQ}}$ again based on the DDT network screened from the DTINet dataset. The only difference here was that we divided the original test set of each independent repeat into three parts, and computed and reported the average evaluation metrics on each part separately. Specifically, the first part (Part 1) includes the samples corresponding to drugs that are in the original test set but not in the training set, and the second part (Part 2) includes the samples corresponding to targets that are in the original test set but not in the training set. The third part (Part 3) includes the samples corresponding to already seen drugs and targets. The numbers of unseen drugs, unseen targets and samples for each independent repeat are in Supplementary Section S4.

**Table 4.** Comparison results of involved methods in the ablation study based on the DTINet dataset

Part	Methods dim: 16	Hits@10	Hits@3	Hits@1	MRR
Part 1	${\mathrm{FLONE}}_{\mathrm{base}}$	0.0596	0.0201	0.0080	0.0313
Part 1	${\mathrm{FLONE}}_{\mathrm{ECFP}-\mathrm{SEQ}}$	**0.4367**	**0.3352**	**0.2379**	**0.3092**
Part 2	${\mathrm{FLONE}}_{\mathrm{base}}$	0.0000	0.0000	0.0000	0.0030
Part 2	${\mathrm{FLONE}}_{\mathrm{ECFP}-\mathrm{SEQ}}$	**0.1872**	**0.1222**	**0.0779**	**0.1167**
Part 3	${\mathrm{FLONE}}_{\mathrm{base}}$	0.6473	0.5589	0.4635	0.5291
Part 3	${\mathrm{FLONE}}_{\mathrm{ECFP}-\mathrm{SEQ}}$	**0.7054**	**0.5978**	**0.5111**	**0.5758**

Part	Methods dim: 128	Hits@10	Hits@3	Hits@1	MRR

Part 1	${\mathrm{FLONE}}_{\mathrm{base}}$	0.1088	0.0363	0.0140	0.0494
Part 1	${\mathrm{FLONE}}_{\mathrm{ECFP}-\mathrm{SEQ}}$	**0.5587**	**0.4528**	**0.3415**	**0.4171**
Part 2	${\mathrm{FLONE}}_{\mathrm{base}}$	0.0000	0.0000	0.0000	0.0028
Part 2	${\mathrm{FLONE}}_{\mathrm{ECFP}-\mathrm{SEQ}}$	**0.2016**	**0.1561**	**0.1146**	**0.1483**
Part 3	${\mathrm{FLONE}}_{\mathrm{base}}$	0.6890	0.6163	0.5522	0.6000
Part 3	${\mathrm{FLONE}}_{\mathrm{ECFP}-\mathrm{SEQ}}$	**0.7650**	**0.6870**	**0.6260**	**0.6723**

*Note*: The bold data indicate the best result under current evaluation metric and data.

The results are provided in Table 4, we observed that ${\mathrm{FLONE}}_{\mathrm{ECFP}-\mathrm{SEQ}}$ clearly out-performed ${\mathrm{FLONE}}_{\mathrm{base}}$ on all three parts. This indicated that introducing similarity-based drug and target encoders into FLONE not only improves its predictions on triples related to seen drugs and targets, but also makes FLONE have the capability to provide effective predictions on ones corresponding to previously unseen drugs and targets. In addition, we further demonstrated the advantages of explicitly considering disease types when inferring targets of the given drug, through an extra experiment detailed in Supplementary Section S5.

### 3.6 Visualization of the embedding spatial layout

**Fig. 3.**
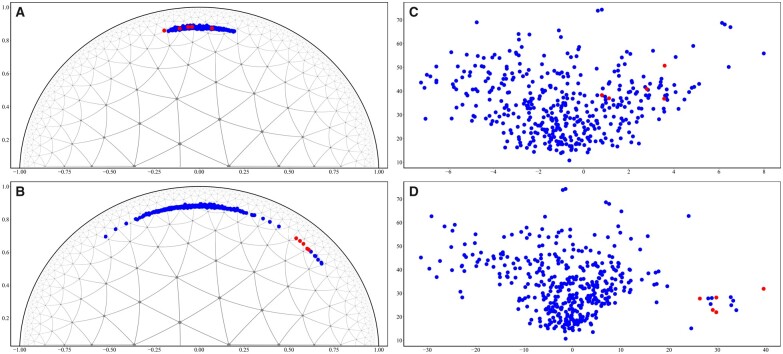
Visualization of the 2D embedding spatial layout of 128-dimension ${\mathrm{FLONE}}_{\mathrm{ECFP}-\mathrm{SEQ}}$ and its Euclidean counterpart. These four layouts display all candidate target embeddings (under the given drug entity *Nitrazepam* and disease relation *leukemia, myeloid, acute*) in different situations (**A–D**). The blue points represent all candidate target embeddings, and the red points represent the embeddings of current positive targets in the test set under the given drug and disease (after dimension reduction). (A) The hyperbolic target embedding layout before applying the translation of the given disease in 2D Poincaré disk. (B) The hyperbolic target embedding layout after applying the translation of the given disease in 2D Poincaré disk. (C) The Euclidean target embedding layout before applying the translation of the given disease in 2D Euclidean space. (D) The Euclidean target embedding layout after applying the translation of the given disease in 2D Euclidean space. Besides, the black arc in layouts (A) and (B) is the boundary of 2D Poincaré disk

To give some insights for showing the captured information by hyperbolic embeddings, we investigated the difference of embedding spatial layout between the Lorentz KGC model and its Euclidean counterpart. Based on the 128-dimension ${\mathrm{FLONE}}_{\mathrm{ECFP}-\mathrm{SEQ}}$ and ${\mathrm{FEC}-\mathrm{FLONE}}_{\mathrm{ECFP}-\mathrm{SEQ}}$ (on DTINet), we projected the Lorentz embeddings and Euclidean embeddings of all candidate target entities (i.e. the targets in the target entity set), for the given drug entity Nitrazepam (*DB01595*) and disease relation *leukemia, myeloid, acute*, to 2-dimension (2D) for visualization (Fig. 3). Specifically, the original Lorentz target embeddings were finally mapped into a 2D Poincaré disk in which the hyperbolic embedding quality can be effectively checked (Balazevic *et al.*, 2019), and the Euclidean ones were mapped to their 2D Euclidean space (the projection details are in Supplementary Section S6).

For the 2D Poincaré disk, as shown in Figure 3A and B, we draw the triangular tiling within it, these triangles had the same size in Euclidean space with the vertices that can be treated as different feature embedding points. We can observe that, the space closer to the boundary of the Poincaré disk can include more embedding points, loosely speaking, there is more capacity to contain more embeddings points and to distinguish them (for downstream tasks) (Balazevic *et al.*, 2019). Through capturing the hierarchical structural information in the DDT network by learning our defined triples, we found that the hyperbolic embeddings of the above targets (from ${\mathrm{FLONE}}_{\mathrm{ECFP}-\mathrm{SEQ}}$) were successfully pushed close to the boundary of the 2D Poincaré disk, and after applying the translation offset of *leukemia, myeloid, acute*, every positive target embedding was clearly separated with all negative target embeddings. For the 2D spatial layout of the corresponding Euclidean target embeddings generated from the Euclidean counterpart of our Lorentz model (Fig. 3C and D), we found that the Euclidean positive target embeddings were not as clearly separated from negative ones as those hyperbolic embeddings. This could indicate that the hyperbolic embedding is able to better capture the DDT triples carrying hierarchical structural information in the DDT networks.

## 4 Conclusion

This article first hypothesizes that, because heterogeneous DDT networks could possess hierarchical structures, the translation-based KGC method could benefit from properly introducing hyperbolic space, which is natural for representing network hierarchies. Within the scope of the more practical target prediction problem—directly ranking all candidate targets for the given drug while explicitly considering disease types, we formulated this problem as a hyperbolic translation-based triple target entity completion task, for testing our hypothesis. We proposed FLONE and evaluated it on two hierarchical DDT networks. Our experimental results showed that, FLONE generates more accurate target predictions than its Euclidean counterparts, which supports our hypothesis. Furthermore, we found that external domain knowledge, such as drug structural and target sequence similarities can be utilized to further improve the predictive accuracy for both seen and previously unseen triples in our framework.

Apart from heterogeneous DDT networks, FLONE could be applied to other complex heterogeneous networks with a hierarchical structure. However, it is worth mentioning that, apart from hierarchical structures, real-world complex networks could exhibit other types of sub-structures, e.g. the cyclic structure. In future work, we plan to introduce more non-Euclidean space, e.g. spherical space specifically for learning cyclic structures into our framework, making it capable of adapting to different network sub-structures. Additionally, FLONE is a network-based method mainly utilizing known DDT network structures to infer unknown associations, therefore exploring how to give a confident prediction to samples, which are totally irrelevant to known DDT triple relationships is also an interesting future direction.

## Supplementary Material

vbad066_Supplementary_Data

## Data Availability

The main data used in this study can be accessed online on GitHub: https://github.com/arantir123/DDT_triple_prediction. The code for reproducing the results presented in our study is available on GitHub: https://github.com/arantir123/DDT_triple_prediction.
