# Supplementary material for: FLONE: fully Lorentz network embedding for inferring novel drug targets
Source: Bioinform Adv. 2023 May 24;3(1):vbad066. doi: 10.1093/bioadv/vbad066 (PMC10235194; doi:10.1093/bioadv/vbad066)
Supplement: vbad066_Supplementary_Data [file vbad066_supplementary_data.docx]

Supplementary Information (SI)

**FLONE: fully Lorentz network embedding for inferring novel drug targets**

Yang Yue, David McDonald, Luoying Hao, Huangshu Lei, Mark S. Butler, and Shan He

Corresponding author: Shan He, Centre for Computational Biology, School of Computer Science, The University of Birmingham, Edgbaston, Birmingham, B15 2TT, UK. Tel: 44-1214142775; Fax: 44-1214144281; E-mail: s.he@cs.bham.ac.uk

**Yang Yue** is a Ph.D. student of the School of Computer Science from the University of Birmingham, UK. His research interests include bioinformatics, machine learning and data mining.

**David McDonald** received the B.Sc., M.Sc., and Ph.D. degrees from the University of Birmingham, UK, in 2015, 2016, and 2020, respectively. He now works for AIA Insights Ltd. as a Chief Technical Officer. His main research interests are drug discovery, complex network analysis, network embedding, heuristic searches and machine learning.

**Luoying Hao** is a Ph.D. student of the School of Computer Science from the University of Birmingham, UK. Her research interests include medical image analysis, video understanding, deep learning.

**Huangshu Lei** is the Chief Scientist of YaoPharma Co., Ltd. His research interests are drug discovery and organic chemistry.

**Mark S. Butler** is the Chief Technical Officer at AIA Insights Ltd. His research interests are drug discovery, natural products and antibiotics.

**Shan He** is an associate professor at the School of Computer Science, the University of Birmingham. His research interests are machine learning, evolutionary algorithms, network medicine and drug discovery.

In the Supplementary Material, we mainly introduce six sections. In section 1, we introduce how to screen drug-disease-target triples based on the Jaccard similarity coefficient. In section 2, we introduce the detailed experimental setup of NeoDTI and KGE_RF when comparing with FLONE. In section 3, we introduce the experiments about fusing the similarity domain knowledge into the Euclidean-based models. In section 4, we introduce the statistics of the unseen drugs, unseen targets, and sample number of each independent test set (mentioned in the manuscript) through tables. In section 5, we introduce an extra experiment to demonstrate the advantages of the triple-wise association prediction. In section 6, we introduce the details about how to project high-dimensional Lorentz and Euclidean embeddings into 2-dimension (2D) Poincaré disk and Euclidean space respectively.

1. **The triple screening based on Jaccard coefficient**

To avoid the implicit data leakage caused by very similar predicates, we perform the following Jaccard-similarity-based [1] screening produce: we first retrieve the drug-target pair set corresponding to every disease, and, for each disease $D_{k}^{'}$, we calculate its Jaccard coefficient with all other diseases based on their corresponding drug-target pair sets, if any of the Jaccard coefficients for $D_{k}^{'}$ are larger than 60%, this disease and corresponding known triples are removed.

1. **Detailed experimental setup of NeoDTI and KGE_RF**

Based on the same original drug-disease-target heterogeneous network extracted from the DTINet dataset and same sample data splitting, we added two representative network-based target prediction methods NeoDTI [8] and KGE_RF [9] into the performance comparison, which were trained on a standard 1:1 sampling of positive and negative samples. As for their testing, they were evaluated in the defined ranking task using original FLONE triple test set (evaluation metrics: MRR etc.), due to that the above methods cannot effectively assign different target probability/ranking scores for the given drug under different diseases, therefore, for each testing drug-disease-target sample, their predicted probability score between the corresponding drug-target pair of the testing triple was used as the final ranking score.

1. **Fusing domain knowledge for the Euclidean-based model**

On the top of the conclusion obtained from the Fusing domain knowledge for FLONE section, to investigate the performance gain of Euclidean translation-based models from importing domain knowledge, based on the best Euclidean model ${FEC-FLONE}_{base}$ (based on the extracted DTINet DDT network and MRR), we did the following experiments, still keeping the same model evaluation settings with the previous sections.

**Table S1.** Comparison results of the involved Euclidean models under 16/128 embedding dimensions based on the DTINet dataset.

| Methods (dim=16) | MRR | Hits@1 | Hits@3 | Hits@10 |
| --- | --- | --- | --- | --- |
| $FEC-\mathrm{FLONE}_{ECFP-SEQ}$ | **0.4537** | **0.3842** | **0.4781** | **0.5958** |
| ${FEC-FLONE}_{ECFP-None}$ | 0.4312 | 0.3694 | 0.4482 | 0.5558 |
| $FEC-\mathrm{FLONE}_{None-SEQ}$ | 0.4145 | 0.3630 | 0.4295 | 0.5161 |
| ${FEC-FLONE}_{base}$ | 0.3608 | 0.3174 | 0.3680 | 0.4484 |
| Methods (dim=128) | MRR | Hits@1 | Hits@3 | Hits@10 |
| ${FEC-FLONE}_{ECFP-SEQ}$ | **0.4759** | **0.4113** | **0.5006** | **0.6025** |
| ${FEC-FLONE}_{ECFP-None}$ | 0.4602 | 0.4004 | 0.4815 | 0.5775 |
| ${FEC-FLONE}_{None-SEQ}$ | 0.4519 | 0.4052 | 0.4627 | 0.5452 |
| ${FEC-FLONE}_{base}$ | 0.4098 | 0.3705 | 0.4139 | 0.4907 |

The bold data indicates the best result under current evaluation metric.

We run the models ${FEC-FLONE}_{ECFP-SEQ}$, ${FEC-FLONE}_{ECFP-None}$, and ${FEC-FLONE}_{None-SEQ}$ on the DTINet DDT network, in which all hyperbolic operations were replaced by corresponding Euclidean ones, and the meaning of subscripts of these model names was consistent with that in the aforementioned section. As shown in Table S1, ${FEC-FLONE}_{base}$ obviously benefited from ECFP6 and target sequence similarity: Compared with ${FEC-FLONE}_{base}$, based on MRR, 25.7% and 16.1% performance improvements were achieved on the dimensions 16 and 128, respectively. However, $\mathrm{FLONE}_{ECFP-SEQ}$ still outperformed ${FEC-FLONE}_{ECFP-SEQ}$, which can further demonstrate the effectiveness of introducing Lorentz space embedding.

1. **Statistics of the independent test sets**

**Table S2.** The statistics of each independent repeat/test set in the comparison experiments.

| DTINet | Repeat 1 | Repeat 2 | Repeat 3 | Repeat 4 | Repeat 5 |
| --- | --- | --- | --- | --- | --- |
| Unseen drug number | 48 | 48 | 56 | 62 | 52 |
| Unseen target number | 41 | 53 | 59 | 53 | 51 |
| BioKG | Repeat 1 | Repeat 2 | Repeat 3 | Repeat 4 | Repeat 5 |
| Unseen drug number | 138 | 148 | 130 | 151 | 149 |
| Unseen target number | 75 | 78 | 79 | 69 | 83 |

**Table S3.** The statistics of sample numbers of the different testing parts in extra ablation study.

|  | Repeat 1 | Repeat 2 | Repeat 3 | Repeat 4 | Repeat 5 |
| --- | --- | --- | --- | --- | --- |
| Part 1 sample number | 3120 | 5533 | 7558 | 6580 | 5609 |
| Part 2 sample number | 3621 | 4658 | 5163 | 4911 | 4862 |
| Part 3 sample number | 26307 | 25040 | 24569 | 24477 | 24562 |

1. **The advantages of the triple-wise association prediction**

We did an experiment to further illustrate the advantages of explicitly considering disease types when inferring targets of the given drug. As illustrated in Introduction, the target and the corresponding given drug could be associated with specific diseases. In other words, given a drug and different diseases, the most associated targets could be different (due to the change of disease types). Formulating this to our ranking task: the best ranking targets of current drug could be different when considering different diseases. For the binary DTI association prediction method, it predicts the pairwise association between drugs and targets, i.e., it will assign the same probability score to a target of the given drug no matter what the associated diseases are. Therefore, explicitly considering the disease type to formulate current target identification task as a triple-wise association ranking/prediction task could bring finer scale virtual screening, allowing models to identify drug targets under particular disease types.

We devised an experiment to further demonstrate this, take drug *DB00363* (*Clozapine*) and *P35348* (*ADRA1A*) as an example, for our DDT network screened from the DTINet dataset, only 265 out of 1160 diseases were recorded to have associations with above DTI pair. Theoretically, the model should assign higher ranking to *P35348* compared with other potential targets of *DB00363* under these 265 diseases, and assign the relatively lower ranking (to *P35348*) under the rest of the diseases (although these diseases may include sparse unidentified positive diseases associated with *DB00363-P35348* pair), instead of assigning the total equal ranking under all diseases. Based on this, we extracted all 1160 triples corresponding to *DB00363*, *P35348*, and the 1160 diseases as the test set. The way of splitting training and validation sets was same to the setting in the Model evaluation settings section (random 6:2 DTI pair-based triple splitting). We recorded the evaluation results of FLONE ($\mathrm{FLONE}_{ECFP-SEQ}$) for the test triples corresponding to the positive diseases and negative diseases separately (Table S4). We found that FLONE did effectively allocate higher ranking for *P35348* under the positive diseases (MRR: 0.9504) and *DB00363*, and allocate low ranking for *P35348* under the negative diseases and *DB00363* (MRR: 0.0964), which further demonstrated the advantages of the triple-wise association predictions.

**Table S4.** The evaluation results on the test drug-disease-target triples corresponding to positive diseases and negative diseases (on DTINet).

|  | Hits@10 | Hits@3 | Hits@1 | MRR |
| --- | --- | --- | --- | --- |
| Positive diseases | 0.9736 | 0.9547 | 0.9396 | 0.9504 |
| Negative diseases | 0.2670 | 0.0603 | 0.0145 | 0.0964 |

1. **The projection details of Lorentz and Euclidean embeddings**

For projecting the high-dimensional Lorentz embeddings to 2-dimensonal (2D), first these Lorentz embeddings are mapped to their isomorphic Poincaré embeddings using the formula (2), where $P^{i}$ is the $i_{th}$ Poincaré embedding, and $L_{s}^{i}$ and $L_{t}^{i}$ are the spatial and time dimensions of corresponding Lorentz embedding $L^{i}$:

|  | $P^{i}=\frac{L_{s}^{i}}{L_{t}^{i}+1}$ | (2) |
| --- | --- | --- |

Then, to project all the high-dimensional Poincaré and Euclidean target embeddings to the corresponding 2D space for visualization, below formulas (3) - (4) by Balazevic, et al. were used, these formulas can keep the original distances and angles of these target embeddings relative to the given drug embedding [2]:

|  | $E_{i}^{x}=\frac{E^{D}}{\left\Vert E^{D} \right\Vert}E_{i},i\in\{D,T_{0},T_{1},\ldots,T_{N}\}$ | (3) |
| --- | --- | --- |
|  | $E_{i}^{y}=\sqrt{\left\Vert E_{i} \right\Vert^{2}-\left\Vert E_{i}^{x} \right\Vert^{2}},i\in\{D,T_{0},T_{1},\ldots,T_{N}\}$ | (4) |

where $E_{i}^{x}$ and $E_{i}^{y}$ are the coordinate on the $x$-axis and $y$-axis of $i_{th}$ new projected embedding, $E^{D}$ is the original high-dimensional embedding of the given drug, $N$ is the number of candidate target entities, and $\left\| \cdot\right\|$ represents the Euclidean norm. After that, the above Lorentz target embeddings have been mapped into a 2D Poincaré disk in which the hyperbolic embedding quality can be effectively checked.

**References**

1. Murphy A H. The Finley affair: A signal event in the history of forecast verification[J]. Weather and forecasting, 1996, 11(1): 3-20.

2. Balazevic I, Allen C, Hospedales T. Multi-relational poincaré graph embeddings[J]. Advances in Neural Information Processing Systems, 2019, 32.
